# TRPV1 Antagonist Prevents Neonatal Sevoflurane-Induced Synaptic Abnormality and Cognitive Impairment in Mice Through Regulating the Src/Cofilin Signaling Pathway

**DOI:** 10.3389/fcell.2021.684516

**Published:** 2021-07-07

**Authors:** Yuqiang Liu, Han Yang, Yifei Fu, Zhenglong Pan, Fang Qiu, Yanwen Xu, Xinping Yang, Qian Chen, Daqing Ma, Zhiheng Liu

**Affiliations:** ^1^Department of Anesthesiology, Shenzhen Second People’s Hospital, The First Affiliated Hospital of Shenzhen University, Shenzhen, China; ^2^Shenzhen Key Laboratory of Neurosurgery, Shenzhen Second People’s Hospital, The First Affiliated Hospital of Shenzhen University Health Science Center, Shenzhen, China; ^3^Division of Anaesthetics, Pain Medicine and Intensive Care, Department of Surgery and Cancer, Faculty of Medicine, Imperial College London, Chelsea and Westminster Hospital, London, United Kingdom

**Keywords:** TRPV1, sevoflurane, synapse, learning and memory, Src, cofilin

## Abstract

Long-term neurodevelopmental disorders following neonatal anesthesia have been reported both in young animals and in children. The activation of transient receptor potential vanilloid 1 (TRPV1) channels in hippocampus adversely affects neurodevelopment. The current study explored the underlying mechanism of TRPV1 channels on long-lasting cognitive dysfunction induced by anesthetic exposure to the developing brain. we demonstrated that TRPV1 expression was increased after sevoflurane exposure both *in vitro* and *in vivo*. Sevoflurane exposure to hippocampal neurons decreased the synaptic density and the surface GluA1 expression, as well as increased co-localization of internalized AMPAR in early and recycling endosomes. Sevoflurane exposure to newborn mice impaired learning and memory in adulthood, and reduced AMPAR subunit GluA1, 2 and 3 expressions in the crude synaptosomal fractions from mouse hippocampus. The inhibition of TRPV1 reversed the phenotypic changes induced by sevoflurane. Moreover, sevoflurane exposure increased Src phosphorylation at tyrosine 416 site thereby reducing cofilin phosphorylation. TRPV1 blockade reversed these suppressive effects of sevoflurane. Our data suggested that TRPV1 antagonist may protect against synaptic damage and cognitive dysfunction induced by sevoflurane exposure during the brain developing stage.

## Introduction

General anesthetic agents are essential to be used to provide a safe and comfortable condition so that complex surgical procedures can be performed. However, there has been an increasing concern that prolonged or repetitious anesthetic exposure may cause long-lasting neurotoxicity and cognitive dysfunction, especially in the young animal and in children (Sanders et al., 2013; Wu et al., 2019). Preclinical studies have shown that exposure early developing brain to commonly used anesthetics may cause certain neurofunctional impairments, such as learning and memory deficits, anxiety-like behaviors and emotional reactivities (Vutskits and Xie, 2016; Colon et al., 2017). However, the underlying molecular and cellular mechanisms are still unclear. Recent childhood cohort studies have shown that a single short exposure to general anesthesia for less than 1 h did not alter neurocognitive functions and behavior (Davidson et al., 2016; Sun et al., 2016; Warner et al., 2018; McCann et al., 2019); However, processing speed and fine motor abilities were decreased after repeated anesthetics exposure (Warner et al., 2018). Thus, the underlying mechanisms of neuronal injury and neurologic dysfunction induced by repeated and prolonged anesthetic exposure deserve further study in the young.

Transient receptor potential vanilloid 1 (TRPV1) is a ligand-gated non-specific cation channel prominently expressed in the dorsal root ganglia (DRG) sensory neurons. This receptor is gated by capsaicin, heat, protons and several exogenous molecules (Alter and Gereau, 2008). TRPV1 has been extensively characterized and is known to play a role in the pain and inflammation processing in the sensory neurons (Palazzo et al., 2010, 2012; Wang Y. et al., 2018). TRPV1 also contributes to neurological diseases, including epilepsy, anxiety, depression and learning and memory disorders (Marsch et al., 2007; Edwards, 2014). In the peripheral nervous system (PNS), certain general and local anesthetics activated and sensitized the TRPV1 channel (Cornett et al., 2008; Leffler et al., 2008), suggesting that this channel may contribute to pain modulation and inflammation in the context of surgery. However, in the central nervous system (CNS), few studies have been conducted on the potential impact of TRPV1 in the developing brain under general anesthesia. Hence, we wonder whether TRPV1 affects the cognitive function in neonatal brain development after anesthetic exposure. In the present study, the effects of sevoflurane, a commonly used inhalational agent, on TRPV1-regulated synaptic density and memory changes together with the underlining molecular mechanisms including the Src/Cofilin signaling pathway were investigated in both cultured mouse hippocampal neuronal cells and C57BL/6 mouse neonates.

## Materials and Methods

### Cell Culture and Treatment

Mouse hippocampal neuronal cell line (HT22 cells) was obtained from the Sun Yat-sen University and cultured as described previously (Liu et al., 2019). Briefly, the cells were maintained in DMEM medium, supplemented with 10% fetal bovine serum (GIBCO BRL, Rockville, MD, United States) and 1% antibiotics (penicillin/streptomycin, 100 U/ml, GIBCO, Waltham, MA, United States), in a humidified incubator containing 5% CO_2_ balanced with air at 37°C. Primary cultures of hippocampal neurons were prepared from the dissociated hippocampus of neonatal mice (<24 h) using a previously described protocol (Liu et al., 2018). The neurons were plated on coverslips coated with Matrigel for at least for 14 days *in vitro* (DIV) before using. The cultures were maintained in a humidified 5% CO_2_ atmosphere balanced with air at 37°C. Both HT22 cells or mouse hippocampal neurons were treated with 4% sevoflurane for 6 h as described by Liu et al. (2019). A selective TRPV1 antagonist, SB 366791 (TOCRIS, Bristol, United Kingdom), was administered to the cell culture medium 1 h with its final concentration of 10 μM before the sevoflurane treatment.

### Experimental Mice

All experimental procedures on mice were approved by the Animal Research Ethics Committee of the Shenzhen Second People’s Hospital and Sun Yat-sen Memorial Hospital. The experiments were performed in these two institutions. C57BL/6 postnatal day seven litter mice with their mothers were obtained from the Guangdong Provincial Laboratory Animal Centre (Guangzhou, China). A single mother and her litters were housed in a cage under a 12 h light-dark cycle at the room temperature of 23 ± 1°C) and 55% humidity. All mice had free access to food and water. Seven-day-old mice of both genders were used for the experiments.

### Grouping and Sevoflurane Exposure

At postnatal day 7 (P7), the litters were randomly divided into four groups (*n* = 10–14/group): (1) Control group (Ctrl); (2) TRPV1 antagonist treatment (SB 366791) group; (3) sevoflurane exposure group (Sev); (4) SB 366791 combined with sevoflurane group (Sev + SB366791). SB 366791 (TOCRIS, Bristol, United Kingdom) was dissolved in DMSO and diluted with normal saline to the appropriate concentration for administration. SB 366791 (500 μg/kg) was injected intraperitoneally 1 h before sevoflurane treatment. Litters were placed in an acrylic chamber and exposed 60% oxygen (balanced with nitrogen) with or without (controls) 3% sevoflurane for 2 h daily for 3 consecutive days as described in previous studies (Lu et al., 2017). During exposure, all litters were kept warm on a pre-heated plate at 37°C. Mice were returned to the housing cages after the treatment. They were allowed to grow for behavioral tests at postnatal day 65 (P65). Another cohorts after treatments were allowed to grow the similar age of those for behavioral tests and then anesthetized using sodium pentobarbital (65 mg/kg, intraperitoneal injection) and sacrificed to harvest brain tissue for further measurements.

### Open Field Test

Mice were placed in the center of a white poly-vinyl chloride apparatus (50 × 50 × 50 cm), and were allowed to continuously locomote for 10 min. The arena was videotaped and analyzed using SMART software (Panlab, Kent, United Kingdom).

### Novel Object Recognition Test (NORT)

Mice were habituated in a square chamber (50 × 50 × 50 cm) with white walls and floor for 10 min on the first day and for 5 min on the second day. The box and objects were cleaned before and between the uses. 24 h after the last habituation, the mice were placed in the chamber with two objects and allowed to freely explore for 5 min “sample phase.” Two hours after the initial exploration, the mice were placed back into the same arena with two objects for 5 min “acquisition phase,” during which one of objects was replaced by a novel object. Exploration counts of each object during two phases were counted. The recognition index was calculated as the percentage of counts spent exploring the novel object over the total exploration counts during the acquisition phase.

### Fear Conditioning Test

The task was performed using a freeze monitor system (San Diego Instruments; San Diego, CA, United States). Background noise level was 65 dB; overhead lighting was used, and 20% ethanol was used as an odor. Mice were placed into a training chamber and allowed to freely explore for 5 min followed by three tone presentations (CS: 5 kHz, 85 dB for 20 s); each tone, was followed by electrical foot-shocks (US: 0.45 mA for 1 s). The interval between three trials was 120 s. Twenty four hours after the training, the mice were placed in the same chamber for 5 min, and freezing behavior was assessed. Forty eight hours later, the mice were tested for freezing responses to the cue. For the cued test, the conditioning chamber was modified as follows: white-walled triangular chamber was replaced with a Plexiglas box, and 2% aloe vera detergent was used as an odor. A dim lamp was used instead of the overhead lighting. Mice were allowed to explore the new environment for 5 min followed by three tones (85 dB, 20 s).

### Immunofluorescent Staining

Hippocampal neuronal cultures and HT22 cells were fixed with PBS containing 4% paraformaldehyde for 1 h at room temperature, washed with PBS, permeabilized with 0.1% Triton X-100 in PBS and blocked in freshly prepared blocking solution (3% donkey serum and 0.2% Triton X-100 in PBS) for 1.5 h at room temperature. The samples were incubated at 4°C overnight with primary antibodies diluted in the blocking solution. After washing with PBS-T (0.2% Triton X-100 in PBS), the samples were incubated with corresponding secondary antibodies for 1 h at room temperature. The following primary antibodies were used (dilution, source): TRPV1 (1:200, NB100-1617, Novus Biologicals, Littleton, CO, United States), MAP2 (1:200, ab5392, Abcam, Cambridge, United Kingdom), synaptophysin (1:500, MAB329, Millipore, Darmstadt, Germany), homer 1 (1:200, 160003, Synaptic Systems, Göttingen, Germany) and Src (1:200, 2110S, Cell Signaling, Beverly, United States).

To visualize surface GluA1, the neurons were fixed with 4% formaldehyde/4% sucrose in PBS. After washing and blocking, surface α-amino-3-hydroxy-5-methyl-4-isoxazolepropionate (AMPA) receptors (AMPARs) were labeled with mouse anti-GluA1 N-terminal antibody (1:200, MAB2263, Millipore, Darmstadt, Germany). Then, the neurons were permeabilized with 0.1% Triton X-100 for 15 min, blocked and incubated with a rabbit anti-GluA1 antibody (1:200, AB1504, Millipore, Darmstadt, Germany) against the intracellular C-terminal domain to stain total GluA1 (tGluA1) at 4°C overnight. After washing with PBS, the neurons were incubated with Alexa Flour-conjugated secondary antibodies. Immunofluorescence images were acquired on a confocal system (LSM 800, Carl Zeiss, Oberkochen, Germany). The images were processed for quantitative analysis using Image-Pro Plus.

To investigate the endosomal distribution of internalized AMPAR in neurons, internalized GluA2 (iGluA2) were labeled and their co-localization with early, recycling and late endosomes was systematically examined using known markers, including early endosome antigen 1 (EEA1), Stx13 and lysosomal-associated membrane protein 1 (LAMP1), respectively, in neurons. Surface GluA2 antibody (extracellular N-terminal domain, Millipore, Darmstadt, Germany) were used to label sGluA2 in the growth medium for 10 min; the cells were rinsed once, stimulated for 2 min with 100 μM AMPA plus 100 μM APV at 37°C. After rinsing, the neurons were returned to the original growth medium for 45 min to allow the internalized AMPAR to recycle back to the cell surface. After washing once with pre-cooled 3% BSA-containing ACSF, the neurons were incubated with non-conjugated mouse secondary antibody at 10°C for 30 min. Then, the neurons were fixed with parafix (4% formaldehyde/4% sucrose/1 × PBS), permeabilized, blocked and incubated with rabbit antibodies against various endosomal markers overnight. Internalized GluA2, endosomal markers and MAP2 were detected using the corresponding secondary antibodies conjugated with Alexa Flour 647, 555, and 488, respectively. Co-localization of internalized AMPAR with each marker was measured as described previously (Lee et al., 2004).

For immunostaining in hippocampal sections, mice were anesthetized with pentobarbital sodium and perfused transcardially with physiological saline, then with 4% formaldehyde. Brains were post-fixed with 4% formaldehyde for 24 h at 4°C, followed by dehydration in 30% sucrose solution for 48 h. Coronal sections from the hippocampus were cut at 10 μm on a cryostat (Leica, Germany), and mounted onto slides. Sections were washed with PBS, and blocked (3% donkey serum and 0.2% Triton X-100 in PBS) for 2 h. Sections were then incubated overnight with anti-synaptophysin (1:500, Millipore, Darmstadt, Germany), followed by secondary antibodies. Immunofluorescence images were acquired on a confocal system (LSM 800, Carl Zeiss, Oberkochen, Germany). The images were processed for quantitative analysis using Image-Pro Plus.

### Synaptosomal Fraction Preparation

Hippocampal tissue samples were homogenized in buffer A (5 mM HEPES, pH 7.4, 1 mM MgCl_2_, 0.5 mM CaCl_2_, 1 mM DTT, and 0.32 M sucrose) containing protease inhibitor and phosphatase inhibitor cocktail (Roche, Mannheim, Germany). The homogenate was centrifuged at 1,400 g for 10 min at 4°C, and the supernatant was discarded. The pellet was resuspended in buffer A and centrifuged at 710 g for 10 min at 4°C to yield supernatant fraction (S1). The S1 was centrifuged again at 13,800 g for 20 min at 4°C. The pellet was resuspended in buffer B (6 mM Tris, pH 8.1, 0.32 M sucrose, 1 mM EDTA, 1 mM EGTA, and 1 mM DTT) containing protease inhibitor and phosphatase inhibitor cocktail. The suspension was used as the crude synaptosomal preparation and analyzed by Western blot.

### Western Blot

The brain tissues were homogenized in RIPA buffer (Beyotime, Shanghai, China) supplemented with protease and phosphatase inhibitors (Roche, Mannheim, Germany) on ice and stored at −80°C until use. Protein concentrations in the supernatant were determined using a BCA assay kit (Beyotime, Shanghai, China). The proteins were separated through the polyacrylamide SDS gels and transferred to 0.45 μm PVDF membrane (Millipore, Darmstadt, Germany). After blocking with 5% non-fat milk, the membranes were incubated with a primary antibody at 4°C overnight. The membranes were then incubated with appropriate HRP-conjugated secondary antibodies (anti-mouse: 1:5,000, 7076S, Cell Signaling; anti-rabbit: 1:5,000, 7074S, Cell Signaling) after washing with TBST (TBS containing 0.2% Tween-20). Then, immunoreactivity was detected by a chemiluminescent reagent (Amersham-GE, Pittsburgh, United States). The following primary antibodies were used (dilution, source): TRPV1 (1:1,000, NB100-1617, Novus Biologicals, Littleton, CO), GAPDH (1:3,000, 2118S, Cell Signaling), GluA1 (1:1,000, AB1504, Millipore), GluA2 (1:1,000, 13607S, Cell Signaling), GluA3 (1:500, MAB5416, Millipore), Src (1:1,000, 2110S, Cell Signaling), p-Src (Tyr416) (1:1,000, 2101S, Cell Signaling), p-Src (Tyr527) (1:1,000, 2105S, Cell Signaling), cofilin (1:1,000, 5175S, Cell Signaling), and p-cofilin (1:1,000, 3313S, Cell Signaling).

### Co-immunoprecipitation

Total protein extracts were obtained from the hippocampal brain tissue of mice. The tissue samples were homogenized in NP-40 lysis buffer (Thermo Fisher Scientific, United States) containing protease and phosphatase inhibitors. The homogenate was incubated on ice for 30 min and then centrifuged at 10,000 g for 10 min at 4°C. The supernatants were immunoprecipitated using a Dynabeads Protein G IP kit (Thermo Fisher Scientific, United States) according to the manufacturer’s instructions. Five micrograms of anti-Src (2110S, Cell Signaling) were used. Western blot was subsequently performed as described above.

### Data Analysis

Data were expressed as the mean ± SEM and analyzed with unpaired student *t*-test and one-way ANOVA followed by *post hoc* Bonferroni test wherever appropriate using GraphPad Prism 7 software (GraphPad Software, La Jolla, California, United States). A *p*-value less than 0.05 was considered to be a statistical significance.

## Results

### Sevoflurane Exposure Reduced Synaptic Density *in vitro* and *in vivo*

**FIGURE 1:**
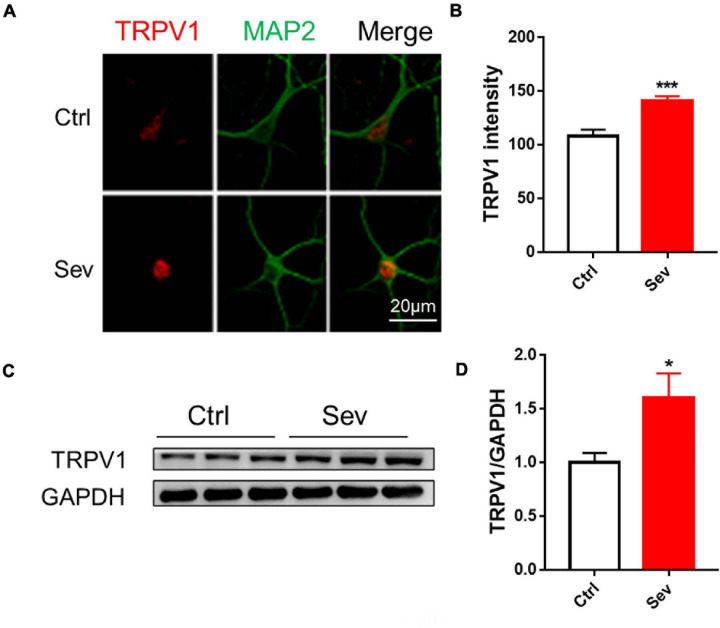
Sevoflurane increased protein levels of TRPV1 channels *in vivo* and *in vitro*. **(A)** Fluorescent images of TRPV1 in hippocampal neurons **(B)** Quantitative analyzed the optical density of TRPV1 protein signal [3 cultures per group, unpaired *t*-test, *t*_(__22__)_ = 4.6, *p* < 0.001]. **(C)** Anesthesia with 3% sevoflurane 2 h daily for 3 days at P7 increased the expression of TRPV1 in the hippocampus of mice harvested at P9 **(D)** Quantification of the Western blot shows that sevoflurane anesthesia increased the protein levels of TRPV1 compared to that observed under the control conditions [*n* = 6, unpaired *t*-test, *t*_(__1__0__)_ = 2.5, *p* = 0.0343]. Data are presented as the mean ± SEM. *p < 0.05; ***p < 0.001. Ctrl, control; Sev, sevoflurane.

Sevoflurane (4%) exposed to primary hippocampal neurons significantly increased TRPV1 expression (Figures 1A,B). The TRPV1 expressions were also increased in the hippocampus following sevoflurane treatment in mice (Figures 1C,D).

**FIGURE 2:**
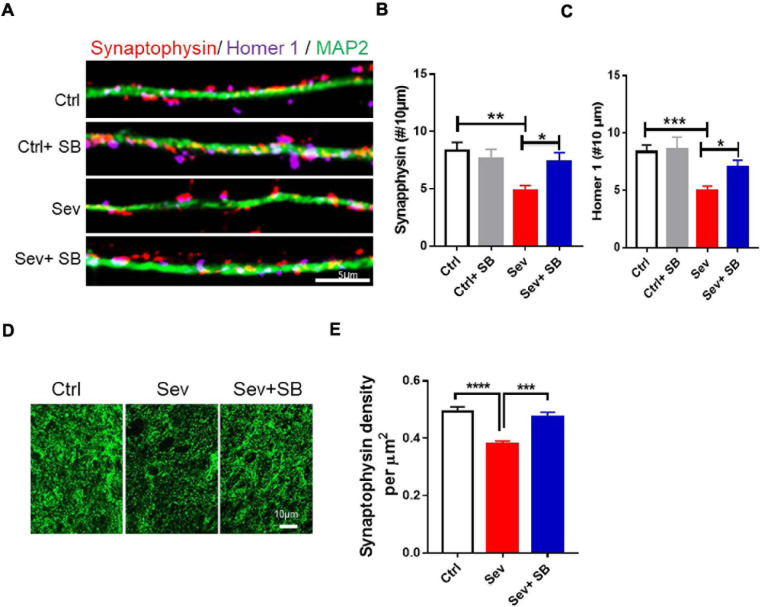
Pretreatment with TRPV1 antagonist SB 366791 significantly suppressed synaptic density loss induced by sevoflurane exposure. **(A)** Fluorescent images of synaptophysin and homer 1 puncta density in hippocampal neurons in the Ctrl and Sev cultures treated with TRPV1 antagonist SB 366791 (10 μM). **(B,C)** Quantitative analysis of synaptophysin **(B)** and homer 1 **(C)** puncta density in the Ctrl and Sev cultures [3 cultures per group, one-way ANOVA, synaptophysin: *F*_(__3_,_56__)_ = 5.0, *p* = 0.0041, homer 1: *F*_(__3_,_58__)_ = 6.9, *p* < 0.001]. **(D,E)** Representative fluorescent images **(D)** and quantitative analysis **(E)** of synaptophysin puncta density in the CA1 area of the with or without SB 366791(500 μg/kg) treatment mice [7 mice per group, *F*_(__2_, _18__)_ = 18.2, *p* < 0.0001]. Data are presented as the mean ± SEM. ANOVA followed by Bonferroni *post hoc* test. *p < 0.05; **p < 0.01; ***p < 0.001; ****p < 0.0001. Ctrl, control; Sev, sevoflurane; SB, SB 366791.

Then, the role of TRPV1 on synaptic density changes was examined after sevoflurane exposure. In the primary hippocampal neuronal cultures at DIV 16 before and after sevoflurane exposure with or without SB 366791, the changes of synaptic density indicated with synaptophysin and homer 1 puncta were significantly decreased by approximate 41 and 40%, respectively, in the sevoflurane-treated cultures compared with the control cultures (Figures 2A–C). Furthermore, pretreatment with SB 366791 for 1 h significantly ameliorated the toxic effects of 4% sevoflurane exposure on neuronal synaptic density (Figures 2A–C). Then, we quantified the synaptic density in the CA1 area in mice. The density of synaptophysin puncta in the Sevoflurane treated mice was significantly reduced by about 24% compared with that in the control mice (Figures 2D,E), whilst SB 366791 reversed this reduction (Figures 2D,E).

### TRPV1 Inhibition Reversed Sevoflurane Exposure Induced Cognitive Function Impairment and AMPAR Delivery Deficiency

**FIGURE 3:**
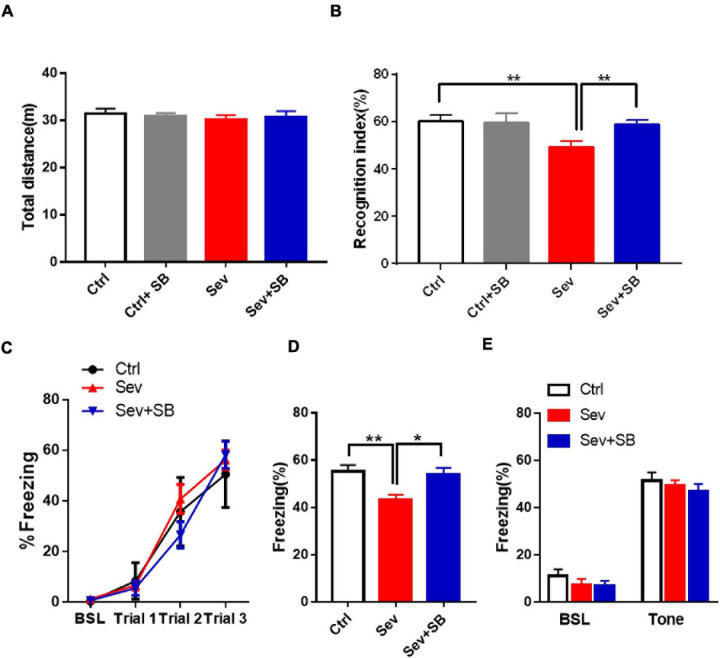
SB 366791 mitigated sevoflurane-induced cognitive impairment in adult mice. **(A)** Total travel distance during 10 min locomotor activity test in the open field test of Ctrl (*n* = 12), Ctrl + 500 μg/kg SB 366791 (*n* = 10), Sev (*n* = 13), and Sev + 500 μg/kg SB 366791 (*n* = 14) mice. **(B)** Recognition index of Ctrl (*n* = 10), Ctrl + 500 μg/kg SB 366791 (*n* = 10), Sev (*n* = 13), and Sev + 500 μg/kg SB 366791 (*n* = 14) mice [one way ANOVA, *F*_(__3_, _43__)_ = 5.8, *p* = 0.0021]. **(C)** Percentage of freezing in Ctrl (*n* = 12), Sev (*n* = 12), and Sev + 500 μg/kg SB 366791 (*n* = 10) mice during fear conditioning training. **(D)** Percentage of freezing in Ctrl (*n* = 12), Sev (*n* = 12) and Sev + 500 μg/kg SB 366791 (*n* = 10) mice during the contextual memory test [one way ANOVA, *F*_(__2_, _31__)_ = 6.9, *p* = 0.0034]. **(E)** Percentage of freezing of Ctrl (*n* = 12), Sev (*n* = 12), and Sev + 500 μg/kg SB 366791 (*n* = 10) mice during the cued test. Data are presented as the mean ± SEM. ANOVA followed by Bonferroni *post hoc* test. *p < 0.05; **p < 0.01. Ctrl, control; Sev, sevoflurane; SB, SB 366791.

Considering that sevoflurane was shown to specifically affect the synaptic puncta by inducing the changes of TRPV1 expression, we determined the effect of TRPV1 on cognitive dysfunction in adulthood induced by sevoflurane exposure of the developing brain. Initially, we assessed the locomotor activity in mice in the open field test. In the mice pre-treated with SB 366791 (SB366791 group), the total distance was similar to that of the wild type mice (Ctrl group) at P65. Additionally, no behavioral deficits were detected in the Sevoflurane with or without SB 366791treated mice (Figure 3A). No differences were found in the center of the arena between any of the groups (data not shown). There were no significant differences in locomotor activity during the 3 training days. In the case of NORT, sevoflurane treated mice had significantly lower recognition index than that in the controls (Figure 3B). Pre-treatment with TRPV1 antagonists SB 366791 abolished this effect of sevoflurane (Figure 3B). Then fear conditioning test, which assesses the hippocampal- dependent and hippocampus-independent memory, was performed. During the training period, all mice exhibited an increase in freezing elicited by the tone and there was no difference between groups (Figure 3C). In the case of the FC contextual test conducted 24 h after the training, sevoflurane treated mice had significantly lower freezing when the animals were place into the same context (Figure 3D), while, SB 366791 abrogated the decline induced by sevoflurane (Figure 3D). However, no significant differences were observed in the FC tone test among the groups (Figure 3E).

**FIGURE 4:**
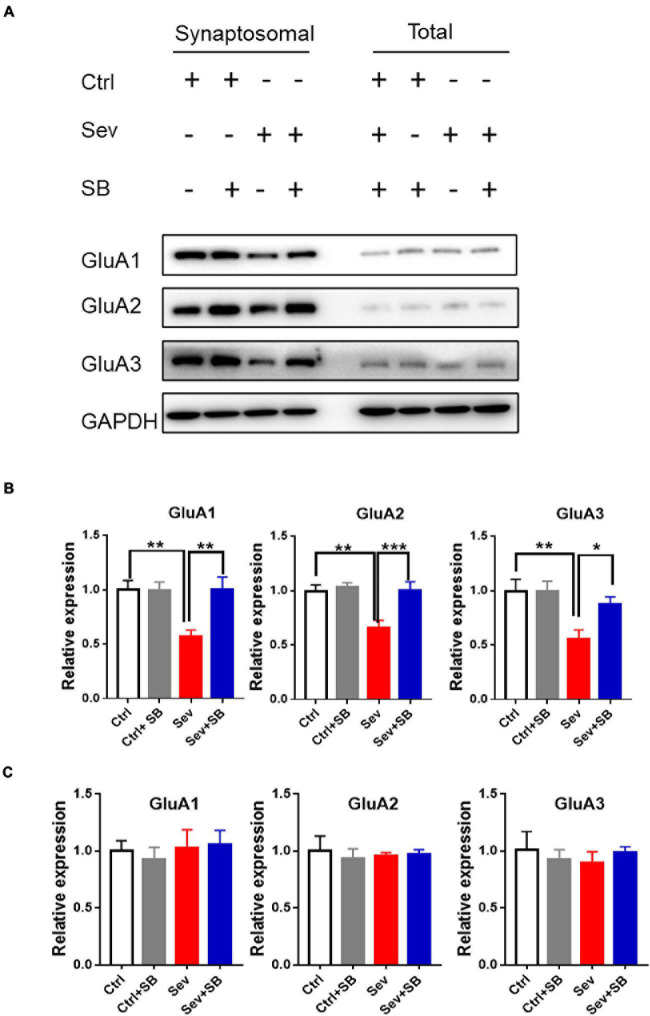
Pre-treatment with TRPV1 antagonist SB 366791 reversed the decrease of the GluA1/2/3 AMPAR subunits caused by sevoflurane exposure. **(A)** Representative Western blots of GluA1/2/3 proteins in crude synaptosomal preparations and total cell lysate obtained from the hippocampal tissue of Ctrl, Ctrl + 500 μg/kg SB 366791, Sev and Sev + 500 μg/kg SB 366791 mice. **(B)** Quantitative analysis of the Western blots [crude synaptosomal, *n* = 8–10 mice per group, one way ANOVA, GluA1: *F*_(__3_, _32__)_ = 6.5, *p* = 0.0015; GluA2: *F*_(__3_, _32__)_ = 7.6, *p* = 0.0006; GluA3: *F*_(__3_, _32__)_ = 6.0, *p* = 0.0023]. **(C)**: quantitative analysis of the Western blots (total protein, *n* = 8–10 mice per group). Data are presented as the mean ± SEM. ANOVA followed by Bonferroni *post hoc* test. *p < 0.05; **p < 0.01; ***p < 0.001. Ctrl, control; Sev, sevoflurane; SB, SB 366791.

**FIGURE 5:**
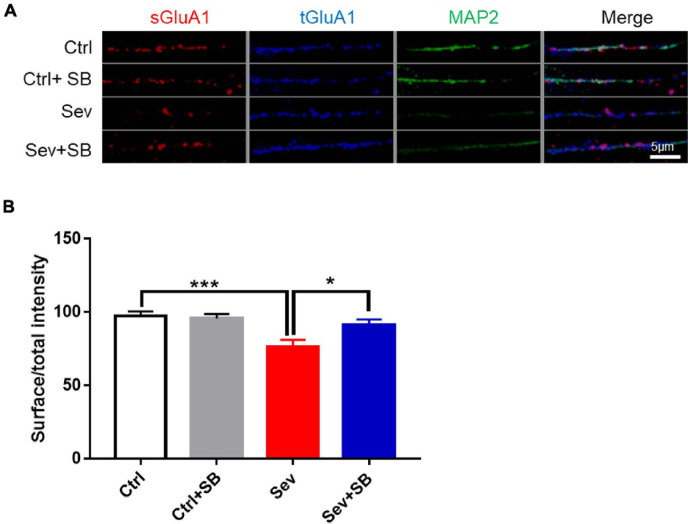
SB 366791 attenuated sevoflurane-induced reduction of surface GluA1 in hippocampus neurons. **(A)** Fluorescent images of surface GluA1 (sGluA1) and total GluA1 (tGluA1) staining in hippocampus neurons from Ctrl and Sev cultures treated with TRPV1 antagonist SB 366791 (10 μM). **(B)** Quantitative analysis of **(A)** [3 cultures per group, one-way ANOVA, *F*_(__3_, _40__)_ = 7.0, *p* = 0.0007]. Data are presented as the mean ± SEM. ANOVA followed by Bonferroni *post hoc* test. *p < 0.05; ***p < 0.001. Ctrl, control; Sev, sevoflurane; SB, SB 366791.

Synaptic AMPA receptor delivery contributes to learning and memory (Mitsushima et al., 2011; Knafo et al., 2012). To test whether TRPV1 is required for changing synaptic AMPAR delivery after sevoflurane exposure, crude synaptosomal fractions from mouse hippocampus were isolated, and both synaptosomal and total AMPAR subunits were detected. Sevoflurane exposure decreased the levels of synaptosomal GluA1, 2 and 3 AMPAR subunits compared to that in the control mice, and SB 366791 maintained the levels of these AMPAR subunits (Figures 4A,B). The levels of AMPAR in the post-nuclear supernatant fraction (total protein) remained similar in all groups of mice (Figures 4A,C). Meanwhile, the surface GluA1 AMPARs in neurons were assessed with immunofluorescence staining. Sevoflurane had a significantly lower surface GluA1 (sGluA1) expression, and pre-treatment with SB 366791 suppressed this reduction induced by sevoflurane exposure (Figure 5).

### TRPV1 Inhibition Reversed Sevoflurane Exposure Induced AMAPR Accumulation in Early and Recycling Endosomes

**FIGURE 6:**
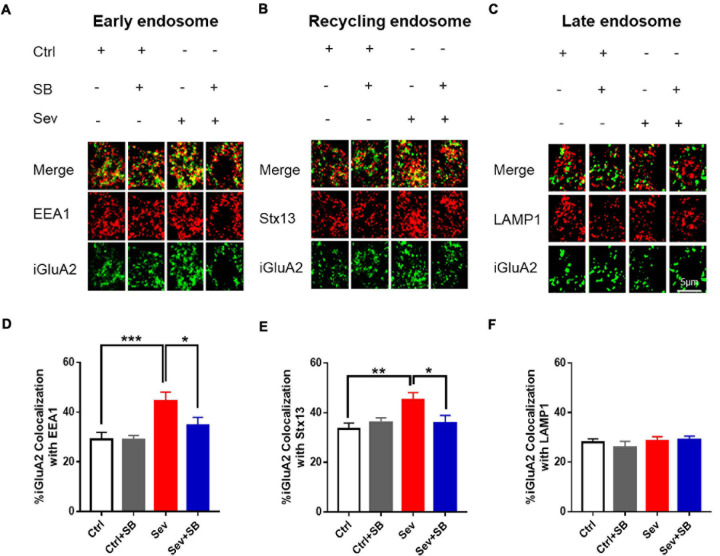
Pretreatment with TRPV1 antagonist SB 366791 reversed the sevoflurane-induced accumulation of internalized AMPAR in endosomes. **(A)** Co-localization of internalized GluA2 (iGluA2, green) and EEA1 (red) in the cell bodies of hippocampal neurons from the Ctrl and Sev cultures treated with TRPV1 antagonist SB 366791 (10 μM). **(B)** Co-localization of iGluA2 and Stx13 (red). **(C)** Co-localization of iGluA2 and LAMP1 (red). **(D)** Quantification of **(A)**. [3 cultures per group, one-way ANOVA, *F*_(__3_, _82__)_ = 6.2, *p* = 0.0008]. **(E)** Quantification of **(B)**. [3 cultures per group, one-way ANOVA, *F*_(__3_, _80__)_ = 3.8, *p* = 0.0129]. **(F)** Quantification of **(C)** [3 cultures per group, one-way ANOVA, *F*_(__3_, _51__)_ = 0.5, *p* > 0.05]. Data are presented as the mean ± SEM. ANOVA followed by Bonferroni *post hoc* test. *p < 0.05; **p < 0.01; ***p < 0.001. Ctrl, control; Sev, sevoflurane; SB, SB 366791.

To determine the cellular mechanism of AMPAR trafficking deficiency in neurons after sevoflurane treatment, internalized GluA2 (iGluA2) was stained to systematically examine its co-localization with early, recycling and late endosomes based on known markers, EEA1, Stx13, and LAMP1, respectively. After sevoflurane treatment, the remaining iGluA2 in neurons showed a significantly higher co-localization with EEA1 and Stx13 than that in the control neurons. SB 366791 ameliorated the increased co-localization of iGluA2 with the EEA1 and Stx13 induced by sevoflurane treatment (Figures 6A,B,D,E). However, the co-localization of iGluA2 with LAMP1 was similar in all group of neurons (Figures 6C,F).

### TRPV1 Interacted With Src and Decreased Cofilin Phosphorylation

**FIGURE 7:**
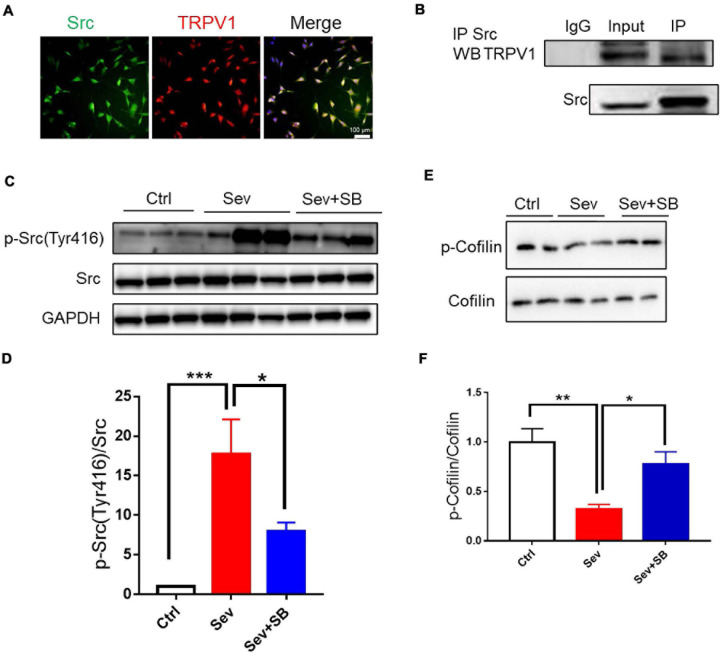
TRPV1 binds to Src and suppresses inhibition of cofilin. **(A)** Representative image of TRPV1 (red) and Src (green) fluorescence in HT22 cell line. **(B)** Co-immunoprecipitation of Src and TRPV1 using anti-Src antibody in hippocampal protein extracts from control mice. **(C,D)** Representative image **(C)** and relative expression of **(D)** Src phosphorylation at tyrosine 416 (p-Src) after with or without SB 366791 (500 μg/kg) treatment [*n* = 6/group, one-way ANOVA, *F*_(__2_, _15__)_ = 10.4, *p* = 0.0015]. **(E)** Representative Western blot bands. **(F)** Quantitative analysis of **(E)** [5 mice per group, one-way ANOVA, *F*_(__2_, _12__)_ = 9.3, *p* = 0.0036]. Data are presented as the mean ± SEM. ANOVA followed by Bonferroni *post hoc* test. *p < 0.05; **p < 0.01; ***p < 0.001. Ctrl, control; Sev, sevoflurane; SB, SB 366791.

Then, the impact of TRPV1 on endosome sorting was analyzed at the molecular level. A non-receptor tyrosine kinase Src is associated with the regulation of cell proliferation and differentiation (Ohnishi et al., 2011). Downregulation of Src can protect mouse brain from injury (Paul et al., 2001; Purcell and Carew, 2003; Liu et al., 2010; Ward et al., 2019). Therefore, we hypothesized that TRPV1 may retain learning and memory by targeting Src cellular signaling. Co-localization of TRPV1 with Src was detected in HT22 mouse hippocampal neuronal cell line (Figure 7A), and co-immunoprecipitation experiments were performed to examine possible interaction between TRPV1 and Src in hippocampal tissue after sevoflurane exposure. The data obtained using mouse hippocampal extract and anti-Src antibody showed that TRPV1 could interact with Src *in vivo* (Figure 7B). The expression of p-Src (Tyr 416) was significantly increased after sevoflurane treatment (Figures 7C,D), and pre-treatment with SB 366791 suppressed this increasement induced by sevoflurane exposure (Figures 7C,D).

Cofilin is an essential regulatory protein with crucial roles in learning and memory through modulating synaptic plasticity and AMPAR mobility (Rust et al., 2010). Previous study reported that cofilin was regulated by Src that triggered cofilin phosphorylation thereby affecting brain function (Wang et al., 2015). Therefore, we hypothesized that TRPV1 may maintain synaptic density and memory by targeting the Src-cofilin pathway. Our results showed that cofilin phosphorylation was significantly reduced in Sev group, while pretreatment with SB 366791 for 1 h before sevoflurane treatment significantly inhibited the reduction of cofilin phosphorylation (Figures 7E,F).

## Discussion

**FIGURE 8:**
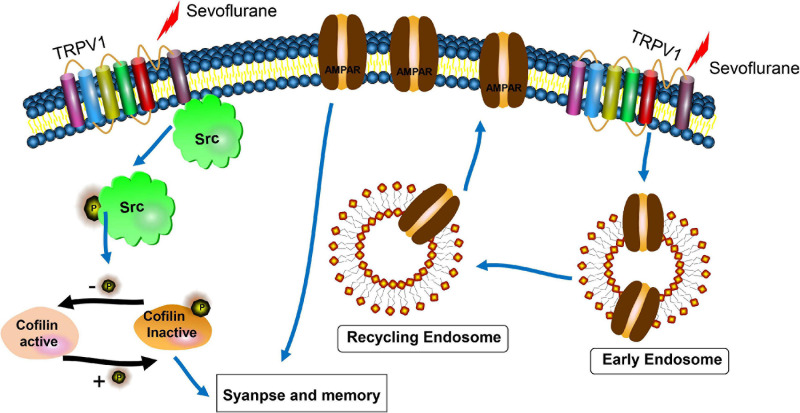
Scheme for the proposed mechanisms of TRPV1 antagonist protection of synaptic loss and memory defects induced by sevoflurane exposure. The underlying mechanisms may be involved with the interactions of TRPV1 and Src, cofilin activation, and AMPAR trafficking deficiency.

In the current study, we demonstrated that repetitious sevoflurane exposure on postnatal day 7 mice led to long-term learning and memory deficits. In neuronal cultures, sevoflurane exposure increased TRPV1 expression, decreased synaptic density, crude synaptosomal and neuronal surface AMPAR expression, as well as defected early and recycling endosomal trafficking in hippocampal neurons. The underlying molecular mechanism may be mediated through an increase in p-Src (Tyr 416) and a decrease in p-cofilin. However, pre-treatment with TRPV1 antagonist SB 366791 before sevoflurane exposure reversed the detrimental effects of sevoflurane both in mice and the hippocampal neurons (Figure 8).

Neurotransmitter receptors, γ-aminobutyrate (GABA) and N-methyl-D-aspartic acid (NMDA) receptors in particular, and other ion channels are molecular targets of general anesthetics (Hemmings et al., 2005), suggesting that multiple anesthetic effects may be associated with various molecular targets in various regions of the nervous system. TRPV1 is a ligand-gated non-specific cation channel responding to various noxious stimuli (Julius, 2013). Previous studies shown that TRPV1 is activated and sensitized by local anesthetics in rodent sensory neurons as well as in HEK293T cells expressing TRPV1 (Leffler et al., 2008). Sevoflurane can sensitize TRPV1 to capsaicin and protons and reduce the threshold for heat activation in nociceptive neurons (Cornett et al., 2008). Our recent data demonstrated that the level of TRPV1 channel was increased in HT22 cells after treated with sevoflurane (Liu et al., 2019). HT22 cells are of neuronal origin; however, these cells may not accurately reflect the mechanisms of the normal neurons; hence, we used primary mouse neurons to investigate the role of TRPV1 in sevoflurane-induced neurotoxicity. The both *in vivo* and *in vitro* data of the present study indicated that the TRPV1 expression in neurons was increased after sevoflurane treatment which in line with our previous study (Liu et al., 2019). Interestingly, another study showed that sevoflurane upregulated the expression of TRPV1 in the airways (Liu et al., 2020). Therefore, all these indicated that sevoflurane can activate this channel in both central nervous system (CNS) and peripheral tissue. However, the underlying mechanism of these effects remains unclear. For instance, sevoflurane may regulate TRPV1 via a ligand-gated mechanism similar to activation of TRP channels by other anesthetics (Matta et al., 2008); the exact mechanisms remain unknown and warrants further study.

Neurons communicate via synapse, and certain changes in synapses are related to a number of brain diseases. We and other (Xiao et al., 2016) reported that synaptic density was reduced after sevoflurane treatment both *in vivo* and *in vitro*. TRPV1 is involved in various functions, including synaptic plasticity in the CNS. Capsaicin, a TRPV1 agonist, upregulated histone deacetylase 2 (HDAC2) resulting in the reduction of synaptic molecules and loss of synaptic density (Wang S. E. et al., 2018). In the present study, TRPV1 antagonist SB 366791 was able to prevent synaptic density decline. Thus, the activation of TRPV1 by sevoflurane reduced synaptic density in hippocampus, and this morphological alteration may subsequently contribute to the impairment of learning and memory.

The causal link between exposure a developing brain to commonly used anesthetics and brain development has not been established and remains controversial despite extensive preclinical studies. Lengthy or repeated exposure of 6–7-day-old rodents to equivalent anesthetics (such as isoflurane, sevoflurane or desflurane) resulted in an impairment of learning and memory in adulthood (Satomoto et al., 2009; Kodama et al., 2011; Ramage et al., 2013; Tao et al., 2016). However, other studies reported that exposure of neonatal non-human primates and rodents to anesthetics did not affect learning and memory (Fredriksson et al., 2007; Zhou et al., 2016). In the present study, exposure of neonatal mice at postnatal day 7–3% sevoflurane 2 h daily for 3 consecutive days resulted in learning and memory dysfunction in the NORT and contextual fear conditioning in adulthood. However, sevoflurane exposure has no effect on tone fear learning. Because the contextual fear conditioning was the hippocampal dependent learning, and tone fear conditioning was hippocampal independent learning (Phillips and LeDoux, 1992; Medina et al., 2002). It is possible that the amygdala function was unlikely impaired and the expression of TRPV1 in the amygdala remained unchanged, thus sevoflurane did not induce memory impairment which was consistent with the previous studies (Ni et al., 2020; Wang et al., 2020).

Published literature suggested that anesthetic exposure to the young and aged animals caused learning and memory disabilities (Dai et al., 2020; Fei et al., 2020; Zheng et al., 2020). In general, adult age animals are resistant to anesthetics-induced neuronal injury although the mechanisms responsible for this difference are unknown. A previous study suggested that extra-synaptic NMDA receptors, which is enriched in the young than the adult age, contributed the sevoflurane induced neurotoxicity (Wang et al., 2016) and this may provide partial explanation. However, adult age animals are not free from neurotoxicity which was also documented previously (Jevtovic-Todorovic et al., 2000). Importantly, under both anesthesia and surgery, the neuronal injuries and hence cognitive impairment in adults were readily detected (Vizcaychipi et al., 2014).

The levels of AMPAR GluA1/2/3 subunits were significantly decreased in the hippocampal synapses of sevoflurane-exposed mice which is similar to the findings of a previous study that pentobarbital and chloral hydrate reduced the expression of cortical and striatal neuronal surface AMPAR (Carino et al., 2012). The various AMPAR GluA subunits were not altered under SB366791 treatment which is not cell toxic (Liu et al., 2019). Previous study has also shown that either upregulation or knockdown of TRPV1 did not affect the expression of GluA1 and GluA2 (Giordano et al., 2012; Du et al., 2020). Thus, SB 366791 itself did not affect the expression of AMPAR. Therefore, sevoflurane decreased the expression of the AMPAR GluA1 subunit in the cultured hippocampal neurons was due to its inherent pharmacological effects, and pre-treatment with TRPV1 antagonist preserved the AMPAR trafficking was likely related to its blocking effect of TRPV1 changes induced by sevoflurane. Considering the results of previous studies, the effect of anesthetics on AMPAR trafficking is unlikely to depend on drug type. A reduction in the number of the receptors in the cell surface pool was accompanied by an increase in the number of the receptors in the intracellular pool. In the current study, the total AMPAR levels were not changed after sevoflurane treatment; thus, changes in the internalization or surface pool would account for AMPAR redistribution. Endosome sorting is the source of AMPA receptor mobilization (Park et al., 2004). Thus, blocking endosome sorting will impact the AMPAR trafficking and subsequent memory impairment. The data of our immunostaining experiments *in vitro* indicated that sevoflurane induced iGluA2 accumulation in the early and recycling endosomes in neurons, and pre-treatment with a TRPV1 antagonist ameliorated this accumulation, suggesting that TRPV1 may be required for AMPAR trafficking. AMPAR trafficking is associated with multiple proteins, including Stx13, Rab11, SNAP47, Rab8, synaptobrevin 2, etc. (Brown et al., 2007; Esteban, 2008; Jurado et al., 2013; Gu et al., 2016). In the present study, inhibition of TRPV1 abolished iGluA2 accumulation in the endosomes, indicating that TRPV1 may interact with endosomal proteins in mice although it warrants further study.

The present study indicated that TRPV1 may interact with Src cellular signaling, and sevoflurane exposure increased the phosphorylation of Src at tyrosine 416. Src can regulate the activities of FAK and cofilin to control neuronal migration (Wang et al., 2015). In p140Cap-knockout mice, over-activation of Src downregulated the RhoA/ROCK/cofilin signaling pathway to impact synaptic plasticity as well as learning and memory (Repetto et al., 2014). Similar to the impairment of the RhoA/ROCK/cofilin pathway induced by Src activation, a decrease in phosphorylation of cofilin was observed in the present study, while SB 366791 reversed this reduction caused by sevoflurane exposure. Brain-derived neurotrophic factor (BDNF) can activate cofilin signaling (Tong et al., 2012); thus, we cannot rule out a possibility that sevoflurane exposure inhibits BDNF to induce the TRPV1-mediated changes in synaptic density and cognitive function. However, our results indicated that the TRPV1 channel and cofilin likely interact each other *per se*.

Our work is not without limitations. First, the both N and C terminals of TRPV1 all contains specific structural domains with slightly different physiological functions; for example, the N-terminal is responsible for a thermal sensor of TRPV1 and channel activity (Yao et al., 2011; Du et al., 2019) whilst C-terminal domains of TRPV1 was reported to be involved in thermo-TRP channel activity, the regulation of voltage-gated channel opening and phosphorylation (Kwak et al., 2000; Goswami et al., 2007; Moiseenkova-Bell et al., 2008; Wang and Chuang, 2011). In our current study, only N-terminal antibodies were used and, therefore, the whole picture changes of TRPV1 are unknown. Second, neonatal mice were only used to study the neurotoxicity of sevoflurane in the current study. Thus, whether the current findings and underlying mechanisms were also evident in adult or even in older age is subjected for future study.

## Conclusion

In conclusion, our results suggested the TRPV1/Src/cofilin signaling pathway likely mediated the abnormalities in synaptic density and neurocognitive function induced by sevoflurane exposure in mice at the brain development stage. These findings may provide a mechanistic foundation for identification of novel therapeutic targets of sevoflurane induced neurotoxicioty.

## Data Availability Statement

The original contributions presented in the study are included in the article/supplementary material, further inquiries can be directed to the corresponding author/s.

## Ethics Statement

The animal study was reviewed and approved by the Committee on the Animal Research Ethics of the Shenzhen Second People’s Hospital and Sun Yat-sen Memorial Hospital.

## Author Contributions

YL and ZL: intellectual ideas and experimental design and manuscript writing. YL, HY, YF, ZP, FQ, and YX: experimental procedures. YL, FQ, XY, and QC: statistical analysis. All authors manuscript editing and revisions.

## Conflict of Interest

The authors declare that the research was conducted in the absence of any commercial or financial relationships that could be construed as a potential conflict of interest.

## Abbreviations

AMPARs: α -amino-3-hydroxy-5-methyl-4-isoxazolepropionate receptors
BDNF: brain-derived neurotrophic factor
CNS: central nervous system
Ctrl: control
DIV: day *in vitro*
DRG: dorsal root ganglia
EEA1: early endosome antigen 1
FC: fear conditioning
GABA: γ -aminobutyrate
GAPDH: glyceraldehyde-3-phosphate dehydrogenase
HDAC2: histone deacetylase 2
LAMP1: lysosomal-associated membrane protein 1
NMDA: N-methyl -D-aspartic acid
NORT: novel object recognition test
P: postnatal day
PNS: peripheral nervous system
Sev: sevoflurane
TRPV1: Transient receptor potential vanilloid 1.

## References

[B1] Alter B. J. Gereau R. W. T. (2008). Hotheaded: TRPV1 as mediator of hippocampal synaptic plasticity. *Neuron* 57 629–631. 10.1016/j.neuron.2008.02.023 18341983

[B2] Brown T. C. Correia S. S. Petrok C. N. Esteban J. A. (2007). Functional compartmentalization of endosomal trafficking for the synaptic delivery of AMPA receptors during long-term potentiation. *J. Neurosci.* 27 13311–13315. 10.1523/JNEUROSCI.4258-07.2007 18045925 PMC6673392

[B3] Carino C. Fibuch E. E. Mao L. M. Wang J. Q. (2012). Dynamic loss of surface-expressed AMPA receptors in mouse cortical and striatal neurons during anesthesia. *J. Neurosci. Res.* 90 315–323. 10.1002/jnr.22749 21932367 PMC3218204

[B4] Colon E. Bittner E. A. Kussman B. McCann M. E. Soriano S. Borsook D. (2017). Anesthesia, brain changes, and behavior: insights from neural systems biology. *Prog. Neurobiol.* 153 121–160. 10.1016/j.pneurobio.2017.01.005 28189740

[B5] Cornett P. M. Matta J. A. Ahern G. P. (2008). General anesthetics sensitize the capsaicin receptor transient receptor potential V1. *Mol. Pharmacol.* 74 1261–1268. 10.1124/mol.108.049684 18689441

[B6] Dai C. L. Li H. Hu X. Zhang J. Liu F. Iqbal K. (2020). Neonatal exposure to anesthesia leads to cognitive deficits in old age: prevention with intranasal administration of insulin in mice. *Neurotox. Res.* 38 299–311. 10.1007/s12640-020-00223-y 32458405

[B7] Davidson A. J. Disma N. de Graaff J. C. Withington D. E. Dorris L. Bell G. (2016). Neurodevelopmental outcome at 2 years of age after general anaesthesia and awake-regional anaesthesia in infancy (GAS): an international multicentre, randomised controlled trial. *Lancet* 387 239–250. 10.1016/S0140-6736(15)00608-X 26507180 PMC5023520

[B8] Du Q. Liao Q. Chen C. Yang X. Xie R. Xu J. (2019). The role of transient receptor potential vanilloid 1 in common diseases of the digestive tract and the cardiovascular and respiratory system. *Front. Physiol.* 10:1064. 10.3389/fphys.2019.01064 31496955 PMC6712094

[B9] Du Y. Fu M. Huang Z. Tian X. Li J. Pang Y. (2020). TRPV1 activation alleviates cognitive and synaptic plasticity impairments through inhibiting AMPAR endocytosis in APP23/PS45 mouse model of Alzheimer’s disease. *Aging Cell* 19:e13113. 10.1111/acel.13113 32061032 PMC7059138

[B10] Edwards J. G. (2014). TRPV1 in the central nervous system: synaptic plasticity, function, and pharmacological implications. *Prog. Drug Res.* 68 77–104. 10.1007/978-3-0348-0828-6_3 24941665

[B11] Esteban J. A. (2008). Intracellular machinery for the transport of AMPA receptors. *Br. J. Pharmacol.* 153(Suppl. 1) S35–S43. 10.1038/sj.bjp.0707525 18026130 PMC2268045

[B12] Fei X. Wang J. X. Wu Y. Dong N. Sheng Z. Y. (2020). Sevoflurane-induced cognitive decline in aged mice: involvement of toll-like receptors 4. *Brain Res. Bull.* 165 23–29. 10.1016/j.brainresbull.2020.08.030 32910992

[B13] Fredriksson A. Ponten E. Gordh T. Eriksson P. (2007). Neonatal exposure to a combination of N-methyl-D-aspartate and gamma-aminobutyric acid type A receptor anesthetic agents potentiates apoptotic neurodegeneration and persistent behavioral deficits. *Anesthesiology* 107 427–436. 10.1097/01.anes.0000278892.62305.9c 17721245

[B14] Giordano C. Cristino L. Luongo L. Siniscalco D. Petrosino S. Piscitelli F. (2012). TRPV1-dependent and -independent alterations in the limbic cortex of neuropathic mice: impact on glial caspases and pain perception. *Cereb. Cortex* 22 2495–2518. 10.1093/cercor/bhr328 22139792 PMC3464411

[B15] Goswami C. Hucho T. B. Hucho F. (2007). Identification and characterisation of novel tubulin-binding motifs located within the C-terminus of TRPV1. *J. Neurochem.* 101 250–262. 10.1111/j.1471-4159.2006.04338.x 17298389

[B16] Gu Y. Chiu S. L. Liu B. Wu P. H. Delannoy M. Lin D. T. (2016). Differential vesicular sorting of AMPA and GABAA receptors. *Proc. Natl. Acad. Sci. U.S.A.* 113 E922–E931. 10.1073/pnas.1525726113 26839408 PMC4763724

[B17] Hemmings H. C. Jr. Akabas M. H. Goldstein P. A. Trudell J. R. Orser B. A. Harrison N. L. (2005). Emerging molecular mechanisms of general anesthetic action. *Trends Pharmacol. Sci.* 26 503–510. 10.1016/j.tips.2005.08.006 16126282

[B18] Jevtovic-Todorovic V. Benshoff N. Olney J. W. (2000). Ketamine potentiates cerebrocortical damage induced by the common anaesthetic agent nitrous oxide in adult rats. *Br. J. Pharmacol.* 130 1692–1698. 10.1038/sj.bjp.0703479 10928976 PMC1572233

[B19] Julius D. (2013). TRP channels and pain. *Annu. Rev. Cell Dev. Biol.* 29 355–384. 10.1146/annurev-cellbio-101011-155833 24099085

[B20] Jurado S. Goswami D. Zhang Y. Molina A. J. Sudhof T. C. Malenka R. C. (2013). LTP requires a unique postsynaptic SNARE fusion machinery. *Neuron* 77 542–558. 10.1016/j.neuron.2012.11.029 23395379 PMC3569727

[B21] Knafo S. Venero C. Sanchez-Puelles C. Pereda-Perez I. Franco A. Sandi C. (2012). Facilitation of AMPA receptor synaptic delivery as a molecular mechanism for cognitive enhancement. *PLoS Biol.* 10:e1001262. 10.1371/journal.pbio.1001262 22363206 PMC3283560

[B22] Kodama M. Satoh Y. Otsubo Y. Araki Y. Yonamine R. Masui K. (2011). Neonatal desflurane exposure induces more robust neuroapoptosis than do isoflurane and sevoflurane and impairs working memory. *Anesthesiology* 115 979–991. 10.1097/ALN.0b013e318234228b 21956042

[B23] Kwak J. Wang M. H. Hwang S. W. Kim T. Y. Lee S. Y. Oh U. (2000). Intracellular ATP increases capsaicin-activated channel activity by interacting with nucleotide-binding domains. *J. Neurosci.* 20 8298–8304. 10.1523/jneurosci.20-22-08298.2000 11069936 PMC6773187

[B24] Lee S. H. Simonetta A. Sheng M. (2004). Subunit rules governing the sorting of internalized AMPA receptors in hippocampal neurons. *Neuron* 43 221–236. 10.1016/j.neuron.2004.06.015 15260958

[B25] Leffler A. Fischer M. J. Rehner D. Kienel S. Kistner K. Sauer S. K. (2008). The vanilloid receptor TRPV1 is activated and sensitized by local anesthetics in rodent sensory neurons. *J. Clin. Invest.* 118 763–776. 10.1172/JCI32751 18172555 PMC2157564

[B26] Liu D. Z. Ander B. P. Xu H. Shen Y. Kaur P. Deng W. (2010). Blood-brain barrier breakdown and repair by Src after thrombin-induced injury. *Ann. Neurol.* 67 526–533. 10.1002/ana.21924 20437588 PMC2919346

[B27] Liu D. Yuan J. Fei X. Zhu Y. Zhou Y. Zhang C. (2020). Effects of inhalation of sevoflurane at different concentrations on TRPV1 in airways of rats at different developmental stages. *Life Sci.* 249:117472. 10.1016/j.lfs.2020.117472 32112870

[B28] Liu Y. Chen C. Liu Y. Li W. Wang Z. Sun Q. (2018). TRPM7 is required for normal synapse density, learning, and memory at different developmental stages. *Cell Rep.* 23 3480–3491. 10.1016/j.celrep.2018.05.069 29924992

[B29] Liu Y. Yang H. Sun C. Wang Z. Liu Z. (2019). Protective effects of TRPV1 inhibition against sevoflurane-induced cell death. *Neurosci. Lett.* 707:134270. 10.1016/j.neulet.2019.05.024 31102705

[B30] Lu H. Liufu N. Dong Y. Xu G. Zhang Y. Shu L. (2017). Sevoflurane acts on ubiquitination-proteasome pathway to reduce postsynaptic density 95 protein levels in young mice. *Anesthesiology* 127 961–975. 10.1097/ALN.0000000000001889 28968276 PMC5685882

[B31] Marsch R. Foeller E. Rammes G. Bunck M. Kossl M. Holsboer F. (2007). Reduced anxiety, conditioned fear, and hippocampal long-term potentiation in transient receptor potential vanilloid type 1 receptor-deficient mice. *J. Neurosci.* 27 832–839. 10.1523/JNEUROSCI.3303-06.2007 17251423 PMC6672910

[B32] Matta J. A. Cornett P. M. Miyares R. L. Abe K. Sahibzada N. Ahern G. P. (2008). General anesthetics activate a nociceptive ion channel to enhance pain and inflammation. *Proc. Natl. Acad. Sci. U.S.A.* 105 8784–8789. 10.1073/pnas.0711038105 18574153 PMC2438393

[B33] McCann M. E. de Graaff J. C. Dorris L. Disma N. Withington D. Bell G. (2019). Neurodevelopmental outcome at 5 years of age after general anaesthesia or awake-regional anaesthesia in infancy (GAS): an international, multicentre, randomised, controlled equivalence trial. *Lancet* 393 664–677. 10.1016/S0140-6736(18)32485-1 30782342 PMC6500739

[B34] Medina J. F. Repa J. C. Mauk M. D. LeDoux J. E. (2002). Parallels between cerebellum- and amygdala-dependent conditioning. *Nat. Rev. Neurosci.* 3 122–131. 10.1038/nrn728 11836520

[B35] Mitsushima D. Ishihara K. Sano A. Kessels H. W. Takahashi T. (2011). Contextual learning requires synaptic AMPA receptor delivery in the hippocampus. *Proc. Natl. Acad. Sci. U.S.A.* 108 12503–12508. 10.1073/pnas.1104558108 21746893 PMC3145714

[B36] Moiseenkova-Bell V. Y. Stanciu L. A. Serysheva I. I. Tobe B. J. Wensel T. G. (2008). Structure of TRPV1 channel revealed by electron cryomicroscopy. *Proc. Natl. Acad. Sci. U.S.A.* 105 7451–7455. 10.1073/pnas.0711835105 18490661 PMC2396679

[B37] Ni C. Qian M. Geng J. Qu Y. Tian Y. Yang N. (2020). DNA methylation manipulation of memory genes is involved in sevoflurane induced cognitive impairments in aged rats. *Front. Aging Neurosci.* 12:211. 10.3389/fnagi.2020.00211 33013350 PMC7461785

[B38] Ohnishi H. Murata Y. Okazawa H. Matozaki T. (2011). Src family kinases: modulators of neurotransmitter receptor function and behavior. *Trends Neurosci.* 34 629–637. 10.1016/j.tins.2011.09.005 22051158

[B39] Palazzo E. Luongo L. de Novellis V. Berrino L. Rossi F. Maione S. (2010). Moving towards supraspinal TRPV1 receptors for chronic pain relief. *Mol. Pain* 6:66. 10.1186/1744-8069-6-66 20937102 PMC2959024

[B40] Palazzo E. Luongo L. de Novellis V. Rossi F. Marabese I. Maione S. (2012). Transient receptor potential vanilloid type 1 and pain development. *Curr. Opin. Pharmacol.* 12 9–17. 10.1016/j.coph.2011.10.022 22104468

[B41] Park M. Penick E. C. Edwards J. G. Kauer J. A. Ehlers M. D. (2004). Recycling endosomes supply AMPA receptors for LTP. *Science* 305 1972–1975. 10.1126/science.1102026 15448273

[B42] Paul R. Zhang Z. G. Eliceiri B. P. Jiang Q. Boccia A. D. Zhang R. L. (2001). Src deficiency or blockade of Src activity in mice provides cerebral protection following stroke. *Nat. Med.* 7 222–227. 10.1038/84675 11175854

[B43] Phillips R. G. LeDoux J. E. (1992). Differential contribution of amygdala and hippocampus to cued and contextual fear conditioning. *Behav. Neurosci.* 106 274–285. 10.1037//0735-7044.106.2.274 1590953

[B44] Purcell A. L. Carew T. J. (2003). Tyrosine kinases, synaptic plasticity and memory: insights from vertebrates and invertebrates. *Trends Neurosci.* 26 625–630. 10.1016/j.tins.2003.09.005 14585603

[B45] Ramage T. M. Chang F. L. Shih J. Alvi R. S. Quitoriano G. R. Rau V. (2013). Distinct long-term neurocognitive outcomes after equipotent sevoflurane or isoflurane anaesthesia in immature rats. *Br. J. Anaesth.* 110(Suppl. 1) i39–i46. 10.1093/bja/aet103 23592692 PMC3695640

[B46] Repetto D. Camera P. Melani R. Morello N. Russo I. Calcagno E. (2014). p140Cap regulates memory and synaptic plasticity through Src-mediated and citron-N-mediated actin reorganization. *J. Neurosci.* 34 1542–1553. 10.1523/JNEUROSCI.2341-13.2014 24453341 PMC6705312

[B47] Rust M. B. Gurniak C. B. Renner M. Vara H. Morando L. Gorlich A. (2010). Learning, AMPA receptor mobility and synaptic plasticity depend on n-cofilin-mediated actin dynamics. *EMBO J.* 29 1889–1902. 10.1038/emboj.2010.72 20407421 PMC2885936

[B48] Sanders R. D. Hassell J. Davidson A. J. Robertson N. J. Ma D. (2013). Impact of anaesthetics and surgery on neurodevelopment: an update. *Br. J. Anaesth.* 110(Suppl. 1) i53–i72. 10.1093/bja/aet054 23542078 PMC3667344

[B49] Satomoto M. Satoh Y. Terui K. Miyao H. Takishima K. Ito M. (2009). Neonatal exposure to sevoflurane induces abnormal social behaviors and deficits in fear conditioning in mice. *Anesthesiology* 110 628–637. 10.1097/ALN.0b013e3181974fa2 19212262

[B50] Sun L. S. Li G. Miller T. L. Salorio C. Byrne M. W. Bellinger D. C. (2016). Association between a single general anesthesia exposure before age 36 months and neurocognitive outcomes in later childhood. *JAMA* 315 2312–2320. 10.1001/jama.2016.6967 27272582 PMC5316422

[B51] Tao G. Xue Q. Luo Y. Li G. Xia Y. Yu B. (2016). Isoflurane is more deleterious to developing brain than desflurane: the role of the Akt/GSK3beta signaling pathway. *Biomed. Res. Int.* 2016:7919640. 10.1155/2016/7919640 27057548 PMC4753322

[B52] Tong L. Prieto G. A. Kramar E. A. Smith E. D. Cribbs D. H. Lynch G. (2012). Brain-derived neurotrophic factor-dependent synaptic plasticity is suppressed by interleukin-1beta via p38 mitogen-activated protein kinase. *J. Neurosci.* 32 17714–17724. 10.1523/JNEUROSCI.1253-12.2012 23223292 PMC3687587

[B53] Vizcaychipi M. P. Watts H. R. O’Dea K. P. Lloyd D. G. Penn J. W. Wan Y. (2014). The therapeutic potential of atorvastatin in a mouse model of postoperative cognitive decline. *Ann. Surg.* 259 1235–1244. 10.1097/SLA.0000000000000257 24263322

[B54] Vutskits L. Xie Z. (2016). Lasting impact of general anaesthesia on the brain: mechanisms and relevance. *Nat. Rev. Neurosci.* 17 705–717. 10.1038/nrn.2016.128 27752068

[B55] Wang J. T. Song L. Z. Li L. L. Zhang W. Chai X. J. An L. (2015). Src controls neuronal migration by regulating the activity of FAK and cofilin. *Neuroscience* 292 90–100. 10.1016/j.neuroscience.2015.02.025 25711940

[B56] Wang S. E. Ko S. Y. Kim Y. S. Jo S. Lee S. H. Jung S. J. (2018). Capsaicin upregulates HDAC2 via TRPV1 and impairs neuronal maturation in mice. *Exp. Mol. Med.* 50:e455. 10.1038/emm.2017.289 29520110 PMC5898893

[B57] Wang S. Chuang H. H. (2011). C-terminal dimerization activates the nociceptive transduction channel transient receptor potential vanilloid 1. *J. Biol. Chem.* 286 40601–40607. 10.1074/jbc.M111.256669 21926175 PMC3220497

[B58] Wang W. Y. Jia L. J. Luo Y. Zhang H. H. Cai F. Mao H. (2016). Location- and subunit-specific NMDA receptors determine the developmental sevoflurane neurotoxicity through ERK1/2 signaling. *Mol. Neurobiol.* 53 216–230. 10.1007/s12035-014-9005-1 25421211

[B59] Wang Y. Gao Y. Tian Q. Deng Q. Wang Y. Zhou T. (2018). TRPV1 SUMOylation regulates nociceptive signaling in models of inflammatory pain. *Nat. Commun.* 9:1529. 10.1038/s41467-018-03974-7 29670121 PMC5906468

[B60] Wang Y. Qian M. Qu Y. Yang N. Mu B. Liu K. (2020). Genome-Wide screen of the hippocampus in aged rats identifies mitochondria, metabolism and aging processes implicated in sevoflurane anesthesia. *Front. Aging Neurosci.* 12:122. 10.3389/fnagi.2020.00122 32457595 PMC7221025

[B61] Ward K. R. Featherstone R. E. Naschek M. J. Melnychenko O. Banerjee A. Yi J. (2019). Src deficient mice demonstrate behavioral and electrophysiological alterations relevant to psychiatric and developmental disease. *Prog. Neuropsychopharmacol. Biol. Psychiatry* 93 84–92. 10.1016/j.pnpbp.2019.02.017 30826459

[B62] Warner D. O. Zaccariello M. J. Katusic S. K. Schroeder D. R. Hanson A. C. Schulte P. J. (2018). Neuropsychological and behavioral outcomes after exposure of young children to procedures requiring general anesthesia: the mayo anesthesia safety in kids (MASK) study. *Anesthesiology* 129 89–105. 10.1097/ALN.0000000000002232 29672337 PMC6008202

[B63] Wu L. Zhao H. Weng H. Ma D. (2019). Lasting effects of general anesthetics on the brain in the young and elderly: “mixed picture” of neurotoxicity, neuroprotection and cognitive impairment. *J. Anesth.* 33 321–335. 10.1007/s00540-019-02623-7 30859366 PMC6443620

[B64] Xiao H. Liu B. Chen Y. Zhang J. (2016). Learning, memory and synaptic plasticity in hippocampus in rats exposed to sevoflurane. *Int. J. Dev. Neurosci.* 48 38–49. 10.1016/j.ijdevneu.2015.11.001 26612208

[B65] Yao J. Liu B. Qin F. (2011). Modular thermal sensors in temperature-gated transient receptor potential (TRP) channels. *Proc. Natl. Acad. Sci. U.S.A.* 108 11109–11114. 10.1073/pnas.1105196108 21690353 PMC3131340

[B66] Zheng F. Fang P. Chang J. Chen M. Zhong Q. Chen T. (2020). Methylene blue protects against sevoflurane-induced cognitive dysfunction by suppressing Drp1 deSUMOylation in aged mice. *Neurochem. Res.* 45 956–963. 10.1007/s11064-020-02976-6 32008150

[B67] Zhou Z. B. Yang X. Y. Tang Y. Zhou X. Zhou L. H. Feng X. (2016). Subclinical concentrations of sevoflurane reduce oxidative stress but do not prevent hippocampal apoptosis. *Mol. Med. Rep.* 14 721–727. 10.3892/mmr.2016.5336 27222114 PMC4918604

